# Computational identification of protein complexes from network interactions: Present state, challenges, and the way forward

**DOI:** 10.1016/j.csbj.2022.05.049

**Published:** 2022-05-27

**Authors:** Sara Omranian, Zoran Nikoloski, Dominik G. Grimm

**Affiliations:** a Technical University of Munich, Campus Straubing for Biotechnology and Sustainability, Bioinformatics, Petersgasse 18, 94315 Straubing, Germany; b Weihenstephan-Triesdorf University of Applied Sciences, Petersgasse 18, 94315 Straubing, Germany; c SynBiofoundry@TUM, Technical University of Munich, Schulgasse 22, 94315 Straubing, Germany; d Bioinformatics, Institute of Biochemistry and Biology, University of Potsdam, 14476 Potsdam, Germany; e Systems Biology and Mathematical Modeling, Max Planck Institute of Molecular Plant Physiology, 14476 Potsdam, Germany; f Technical University of Munich, Department of Informatics, Boltzmannstr. 3, 85748 Garching, Germany

**Keywords:** Protein Complex Prediction, Protein-Protein interaction network, Network Clustering Algorithms, Network embedding

## Abstract

Physically interacting proteins form macromolecule complexes that drive diverse cellular processes. Advances in experimental techniques that capture interactions between proteins provide us with protein–protein interaction (PPI) networks from several model organisms. These datasets have enabled the prediction and other computational analyses of protein complexes. Here we provide a systematic review of the state-of-the-art algorithms for protein complex prediction from PPI networks proposed in the past two decades. The existing approaches that solve this problem are categorized into three groups, including: cluster-quality-based, node affinity-based, and network embedding-based approaches, and we compare and contrast the advantages and disadvantages. We further include a comparative analysis by computing the performance of eighteen methods based on twelve well-established performance measures on four widely used benchmark protein–protein interaction networks. Finally, the limitations and drawbacks of both, current data and approaches, along with the potential solutions in this field are discussed, with emphasis on the points that pave the way for future research efforts in this field.

## Graphical abstract

**Figure:**
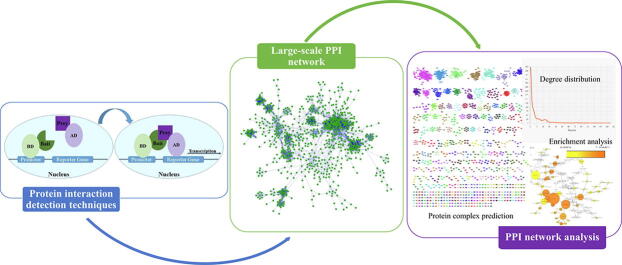

## 1 Introduction

Proteins are essential components of all living organisms and are composed of a polypeptide chain of amino acids that translates information encoded in genes. The three-dimensional shape of a protein is described by its tertiary structure. The protein ternary structure enables specific chemical groups to be placed at exact positions in a three-dimensional space, leading to particular enzymatic functions and other important structural, transport, and regulatory functions in an organism [29]. However, most proteins do not function as a single entity. Instead, they often interact with other proteins to form large macromolecules that coordinate and perform diverse molecular functions within the cell [75], [100].

Protein-protein interactions (PPI) have different structural characteristics which are related to their physiological function and evolution [67]. If an interaction occurs between two or more identical polypeptide chains, it is referred to as a homo-oligomeric complex. In contrast, if the interaction involves two or more non-identical chains, it leads to a hetero-oligomeric complex [56]. In addition to composition, other types of complexes are distinguishable according to whether they are obligate or non-obligate. In a non-obligate complex, a protein forms a stable well-folded structure without any assistance from other proteins. However, some proteins cannot make a stable well-folded structure themselves and form protein complexes to stabilize the constituent proteins, leading to obligate protein complexes [2].

Furthermore, protein complexes can be grouped into transient and permanent based on their lifespan. The PPIs of transient complexes are established and resolved transiently, whereas the PPIs of permanent complexes are stable. Interestingly, most obligate protein complexes are also permanent; however, the non-obligate complexes can be permanent or transient [2]. For instance, hemoglobin is an important permanent protein complex composed of four globular protein subunits [65]. In multicellular organisms, cells must communicate with other cells by forming transient protein complexes, e.g. during cell signaling to transfer information [20]. It is important to note that not all PPIs and protein complexes fall into distinct categories.

Proteins are often involved in more than one complex in different subcellular compartments and biological processes. Therefore, it is important to accurately identified protein complexes to understand not only protein complex formations but also the higher-level cellular organization [25]. Several techniques are available to determine protein complexes, which can be categorized into: (i) experimental and (ii) computational techniques. Tandem Affinity Purification and Mass Spectrometry (TAP-MS) [70] is one of the most commonly used experimental methods to reveal a global map of the complexome (i.e. the set of protein complexes in a cell) for different species [42], [33]. Nevertheless, the protein complexes from TAP-MS are incomplete and reliable only to a certain degree due to the in-built technical biases [25]. The advent of high-throughput techniques, such as: yeast two-hybrid (Y2H) [96] and affinity purification mass spectrometry (AP-MS), have facilitated the assembly of genome-wide protein–protein interaction (PPI) data for several model organisms [34], [68]). These datasets have enabled the study and identification of protein complexes computationally, through mapping interaction data into network representations. In these networks, individual proteins serve as nodes and their interactions as edges [26], [98]. It should be noted that the current state-of-the-art high-throughput techniques produce a considerable proportion of spurious interactions, which results in false-positive as well as false-negative interactions in PPI networks [85], [6]. Therefore, computational approaches should consider the effect of noisiness and incompleteness of PPI data to enable the prediction of more accurate protein complexes. To consider the effect of false-positive interactions, the quality of PPIs needs to be assessed by assigning a confidence score (i.e. affinity) to each interaction that reflects the reliability of the inferred interactions [17], [18], [44]. The interactions with low confidence values may, in turn, be discarded in subsequent analyses. On the other hand, link prediction algorithms [10], [41] and different local and global network topological metrics can be employed to score false-negative interactions and insert the high-scored ones to the original PPI networks as a result [59].

Although experimental data contain biological and technical noise, that may lead to false-positive and false-negative interactions, several computational approaches have been proposed to moderate these limitations by efficiently analyzing a large amount of data to predict protein complexes. Consequently, several surveys [77], [15], [97] have reviewed and summarized existing computational approaches by comparing and evaluating their performance on available PPI networks. While these studies cover only approaches until 2016, with this review, we aim to systematically compare and contrast the state-of-the-art approaches that have been proposed within the last two decades, from 2002 until 2022.

**Fig. 1:**
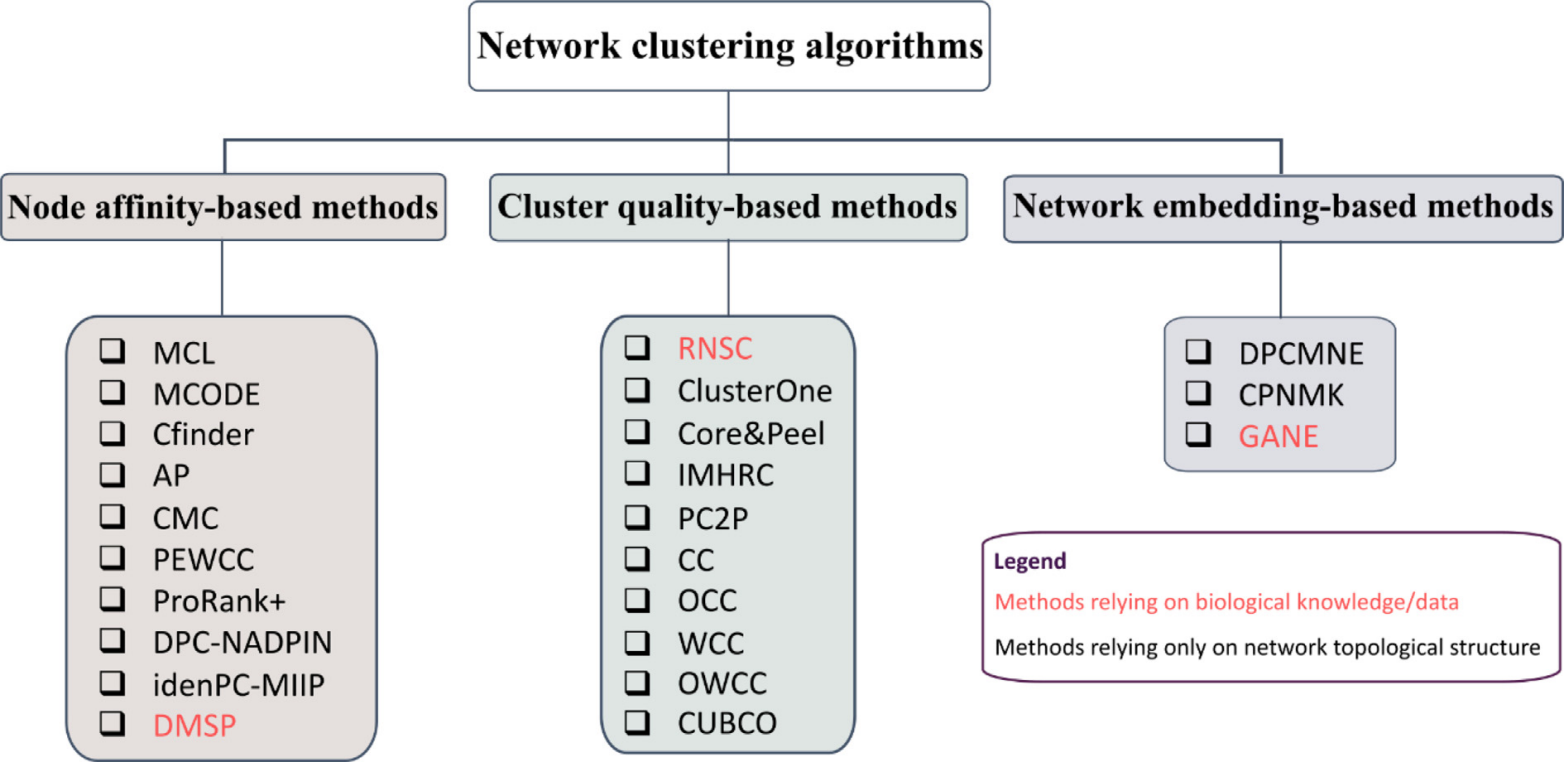
**Categories of the network clustering algorithm used in the protein complex prediction with PPI networks.** The network clustering algorithms require as input either only a PPI network (methods in black color) or both on PPI network and biological information (methods in red color). Regardless of the input, the existing network clustering algorithms with applications to complex prediction can be divided into three categories, namely: node affinity-based, cluster quality-based, and network embedding-based methods. For each category, several examples are given and explained in this review. (For interpretation of the references to color in this figure legend, the reader is referred to the web version of this article.)

In this study, before going through the current approaches, we first introduce important terminologies in this field. We then provide a comprehensive and updated review of various state-of-the-art computational methods in the field of protein complex identification. The computational methods are organized into three categories, namely: (i) cluster-quality-based methods, (ii) node-affinity-based methods, and (iii) network embedding methods (see Fig. 1). Furthermore, we will discuss the advantages and disadvantages of the methods in these three categories, followed by an evaluation of the performance of 18 state-of-the-art approaches from the three categories on four PPI networks. Finally, the bottleneck problems and their potential solutions in this important field will be discussed.

## 2 Graph-theoretic concepts

Let G=(V,E) be a simple graph with a set of nodes V and edges E. A weighted graph G=(V,E,w) is a graph, in which each edge is assigned a weight, specified by the function $w:E\to R_{+}$, that quantifies the affinity for interactions between the two end nodes (i.e. proteins). Graph G is connected if there is a path between every pair of nodes; otherwise, it is disconnected. Nodes u and v are neighbors if there is an edge between them. An adjacency matrix is a squared matrix such that its elements indicate whether pairs of nodes are neighbors or not in the graph. The nodes in a line graph of a graph G corresponds to the edges of G; the nodes in the line graph are adjacent if the corresponding edges in G are adjacent. A graph G is complete (i.e. clique) if for every pair of nodes u,v∈V in the graph, there exists an edge (u,v). A graph is called bipartite if the node set can be divided into two disjoint partitions M and N, such that every edge connects a node in M to one in N. A complete bipartite graph (i.e. biclique) is a special kind of bipartite graph where every node of M is connected to every node of N with an edge. A biclique spanned graph is a biclique that may include additional edges between the nodes in each partition. The density of a graph G indicates to what extent G differs from a clique; this is quantified by the ratio of the number of edges in G and the maximum possible number of edges in the graph on the same number of nodes. The shortest path is a path between two nodes in a graph for which the sum of edge weights between two nodes is minimized.

## 3 PPI networks and gold standards of protein complexes

There are already several databases of PPIs across different model organisms. However, it is worth noting that regardless of which technique is used, the molecular interaction data may contain artifacts due to their design [93], multiple stages of washing in their purification step [47], or the cell lysis step [80], [74], to name a few.

The Database of Interacting Proteins (DIP) [90] collects experimentally verified PPIs from scientific articles for different species. A general repository for interaction datasets (BioGRID) [78] is another database that is similar to DIP and includes interactions through comprehensive curation of experimentally reported PPIs. On the other hand, the STRING database [79] integrates both, experimentally and computationally reported PPIs and assigns a score to an interaction based on available evidence.

There exist other datasets that are species-specific and for simplicity, these PPI networks are just named after the corresponding first author. Gavin [24], Collins [17], [18], Krogan Core, and Krogan Extended [42] are commonly used as PPI networks for S. cerevisiae. These PPI networks are edge-weighted and were obtained experimentally. The weights (in the range between zero and one) denote the reliability of each interaction. The interaction weights in the Collins PPI network are based on the purification enrichment score, while in the Gavin PPI network, the weight indicates the socio-affinity index which calculates the log-odds of how many times pairs of proteins are observed together as preys, or bait and prey in the network. The interactions in the Krogan PPI network are weighted based on the integration of mass spectrometry scores. Moreover, Babu [5] and Cong [19] are two *E. coli* PPI networks. The former is obtained experimentally from affinity purification mass spectrometry (AP-MS), while the latter contains interactions that are predicted by utilizing evolutionary signatures in protein sequence and structure. Finally, PIPs [52] is a database of predicted H. sapiens PPI networks based on a naïve Bayes classifier [72]. The key graph-theoretic properties of the aforementioned PPI networks can be found in Table 1.

**Table 1:** Summary of protein–protein interaction networks.

Name	Version / update date	Species	#Proteins	#Interactions
DIP [90]	5/Feb/2017	All	28,255	76,881
BioGRID [78]	4.4.206	All	80,939	1,191,174
STRING [79]	11.5	All	67.6 mio	>20 bln
Babu [5]	27/Nov/2017	*E. coli*	2,045	12,801
Cong [19]	12/Jul/2019	*E. coli*	1,476	1,618
Collins [17], [18]	Mar/2007	S. cerevisiae	1,622	9,074
Gavin [24]	Jan/2006	S. cerevisiae	1,855	7,669
Krogan [42]	Marc/2006	S. cerevisiae	6,380	21,440
PIPs [52]	v1.1	H. sapiens	5,751	79,441

Besides PPI networks, different sets of protein complexes are available as gold standards. CYC2008 [66], an update to the Munich Information Centre for Protein Sequences (MIPS) catalog [54], and complexes derived from the *Saccharomyces* Genome Database (SGD) [31] are the most common protein complex reference sets for S. cerevisiae. These contain protein complexes that are verified in small-scale experiments. Furthermore, CORUM [27] provides a reference set of manually annotated protein complexes from mammalian organisms. Finally, the EcoCyc [37] and Metabolic (Met) [40] reference sets include manually curated protein complexes and complexes based on genome-scale metabolic networks, respectively. An overview of different gold standards of protein complexes is shown in Table 2.

**Table 2:** Summary of protein complex gold standards.

Name	Species	#Proteins	#Complexes	#Complexes ≥ 3
CYC2008 [66]	S. cerevisiae	1,627	408	236
SGD [31]	S. cerevisiae	1,279	323	238
CORUM [27]	H. sapiens	4,479	4,274	2,783
EcoCyc [37]	*E. coli*	749	299	181
Met [40]	*E. coli*	475	206	118

## 4 Computational prediction of protein complexes from PPI networks

Several computational approaches have been developed to identify the underlying protein complexes and functional modules in PPI networks. They arise as a complementary tool next to experimental techniques to enhance the existing recourses and knowledge by finding novel protein interactions and complexes. However, due to the highlighted limitations of experimental screens, the performance of computational approaches is restricted. These limitations convey three main challenges of computational methods for protein complex predicton: (1) difficulties in detecting sparse complexes; (2) difficulties in detecting small complexes constituting of two or three proteins; (3) difficulties in detecting overlapping complexes, i.e. the complexes that share one or many proteins [76]. Moreover, most of the existing approaches depend on several parameters, which complicate the interpretation of the predicted protein complexes. The latter is due to the need to identify the best parameter values for every combination of PPI networks, gold standards, and performance measures (see Section 5 “Evaluation metrics”)—a challenging and practically infeasible task. Consequently, different values for the parameters may result in different sets of predicted protein complexes.

**Fig. 2:**
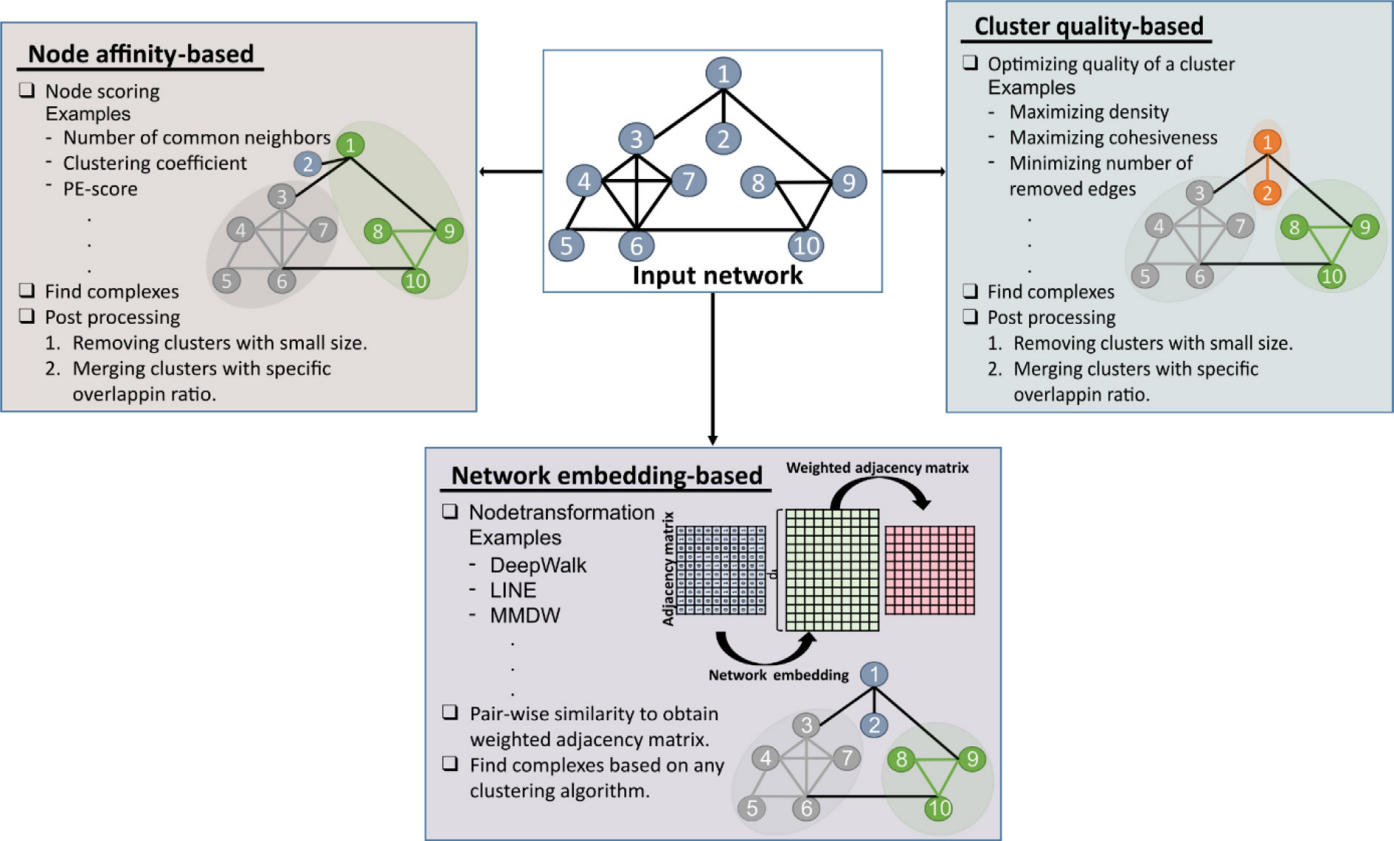
**Categories of computational approaches to detect protein complexes.** Node affinity-based approaches use different node scoring methods, while cluster quality-based approaches cast the protein complex prediction as an optimization problem on PPI networks. However, the next steps to find protein complexes are almost the same for both categories. The network embedding-based approaches predict protein complexes, first by transforming each node to a vector, which is followed by finding similarities between pairs of node vectors. Lastly, they utilize any network clustering algorithms to find protein complexes.

The computational approaches can be categorized in several ways. Some methods rely solely on PPI networks, whereas others depend on additional biological information. The methods in the latter category, such as Dense neighborhood Extraction using Connectivity conFidence Features (DECAFF) [46] and Restricted Neighborhood Search Clustering (RNSC) [39], utilize functional information and gene ontology data to predict protein complexes. The methods that use the network topology to find densely connected components are known as community detection methods or graph clustering algorithms in graph theory. Further, graph clustering algorithms can be organized into three subcategories according to their methodology: (i) node affinity-based methods; (ii) cluster quality-based methods, and (iii) network embedding methods (Fig. 2, Table 3).

**Table 3:** **Overview of computational approaches for prediction of protein complexes from PPI networks.** The current state-of-the-art methods are divided into three categories: node-affinity, cluster-quality, and network embedding-based approaches. The input of each method is shown in the second column. A link to the public implementation of each method (if available) along with the year of publication is given in the third column. Other properties such as the number of parameters, the capability of the method to use edge-weights or to predict overlapping protein complexes are given in the last three columns, respectively.

Category	Biological Knowledge/data	Method – Website	Feature(s)
Node Affinity-based approaches	×	MCL [2002]	MCL has 2 parameters and utilizes edge weights. It detects non-overlapping clusters. The size of the clusters depends on the inflation parameter.
		MCODE [2003]	MCODE depends on 5 parameters and does not utilize the edge weights. By setting the fluff parameter, it can detect overlapping clusters. The predicted clusters are of high density. MCODE is unable to find sparse clusters.
		CFinder [2006]	CFinder has 2 parameters and employs edge weights. The predicted clusters have a clique topology. CFinder detects overlapping clusters, while it is unable to find sparse ones.
		AP [2007]	AP has 1 parameter, that affects the cluster formation, and it does not use edge weights. It detects non-overlapping and dense clusters.
		CMC [2009]	CMC has 2 parameters and employs edge weights. The clusters have a clique topology. CMC is unable to find sparse clusters. The size of the clusters depends on the parameters. CMC can detect overlapping clusters.
		PEWCC [2013]	PEWCC has 2 parameters and uses edge weight. It deals with false-positive interactions by introducing a PE-score, while it does not consider the effect of false-negative ones. PEWCC detects highly overlapped and repetitive clusters.
		ProRank + [2014]	ProRank + has 2 parameters and employs edge weights. It considers the effect of false-positive interactions but not the false-negative ones. ProRank + detects overlapping clusters.
		DPC-NADPIN [2016]	DPC-NADPIN has 2 parameters and does not utilize edge weights. It incorporates gene expression data to create a dynamic PPI network. It is unable to predict small clusters. DPC-NADPIN detects overlapping clusters.
		idenPC-MIIP [2020]	idenPC-MIIP has 2 parameters and employs edge weights. It considers the effect of false-positive interactions by calculating MIIP-score. idenPC-MIIP can detect overlapping clusters.
	Microarray data		
	DMSP [2007]	DMSP depends on 2 parameters. It considers the effect of false-positive edges by calculating the gene-expression similarity between pairs of protein. DMSP can predict non-overlapping clusters.	
Cluster quality-based approaches	×	miPALM [2010]	miPALM has 2 parameters and assigns edge-weights. It detects dense clusters and is unable to predict small and sparse clusters. miPALM predicts overlapping clusters; however, it does not consider the effect of false-positive and false-negative interactions.
		ClusterOne [2012]	ClusterOne has 3 parameters and it utilizes edge weights. It is unable to find small and sparse clusters. ClusterOne predicts overlapping clusters; however, it does not consider the effect of false-negative interactions.
		Core&Peel [2016]	Core&Peel depends on 3 parameters and it uses the edge weights. It predicts dense complexes. The size and density of the clusters depends on 2 parameters. Core&Peel can detect overlapping clusters; however, it does not consider the effect of false-negative interactions.
		IMHRC [2017]	IMHRC has 5 parameters and it employs edge weights. It is unable to find small and sparse clusters. IMHRC can detect overlapping clusters; however, it does not consider the effect of false-negative interactions.
		PC2P [2020]	PC2P is a parameter-free algorithm. It can detect small and large as well as sparse and dense clusters. However, it does not utilize edge weights, but can detects non-overlapping clusters.
		CC [2021]	CC is a parameter-free approach. It can detect small and large as well as sparse and dense clusters. However, it does not utilize edge weights, and can detect non-overlapping clusters.
		OCC [2021]	OCC is a parameter-free approach. It can detect small and large as well as sparse and dense clusters. Although it does not utilize edge weights, it can detect overlapping clusters.
		WCC [2021]	WCC is a parameter-free approach. It can detect small and large as well as sparse and dense clusters. While it utilizes edge weights, it can detect non-overlapping clusters.
		OWCC [2021]	OWCC is a parameter-free approach that uses edge weights. It can detects small and large as well as sparse and dense clusters. OWCC detects overlapping clusters, however it does not consider the effect of false-negative interactions.
		CUBCO [2022]	CUBCO is a parameter-free approach that uses edge weights. It can detect small and large as well as sparse and dense clusters. CUBCO considers the effect of false-negative as well as false-positive interactions; however, it cannot detect overlapping clusters.
	Functional homogeneity	RNSC [2004]	RNSC depends on 7 parameters and it does not consider edge weights. RNSC is a randomized algorithm and in each round, it generates different clusters. It is highly dependent on the initial clusters and it is unable to detect overlapping clusters.
Network embedding-based approaches	×	CPNM [2020]	CPNM has 6 parameters and uses edge weights. It finds non-overlapping clusters. CPNM detects dense clusters and not sparse ones.
		DPCMNE [2021]	DPCMNE is dependent on 5 parameters and uses the edge weights. It is not able to detect sparse clusters, but it can detect overlapping clusters.
	Gene Ontology	GANE [2018]	GANE has 3 parameters and it utilizes edge weights. While it cannot detect sparse clusters, it is able to predict overlapping clusters.

## 5 Node affinity-based methods

Node affinity-based approaches consider the inherent relationship among nodes during the process of identifying clusters. The clusters are generated from seeds and expanded by nodes in their neighbor with a high affinity score.

The Markov CLustering algorithm (MCL) is one of the most widely used graph clustering algorithms [22]. The MCL simulates a flow in PPI networks using two steps: expansion and inflation. The expansion parameter allows the flow to connect different regions of the graph, whereas the inflation parameter is used for strengthening and weakening the flow that tunes the granularity of the clusters. Therefore, the size of the clusters is highly dependent on the inflation parameter. The algorithm repeats the expansion and inflation procedure until convergence and then the PPI network is partitioned into non-overlapping clusters.

The Molecular COmplex DEtection (MCODE) method is a heuristic approach and works based on local neighbor densities [7]. It has three main steps: (1) node scoring, (2) molecular complex prediction, and (3) post-processing. In the first step, it assigns a score to each node based on the density of the node neighborhood. Next, starting from a node with the highest score, a protein complex is grown iteratively. The depth limit parameter controls how far the growth should be continued to form a protein complex, while the vertex weight percentage parameter regulates the score differences between nodes within the complex. Finally, MCODE has two post-processing steps: fluffing and haircut. In the former, the complex will be expanded by other nodes that interact with many nodes of the same complex. In the latter one, the nodes with only a single interaction with the rest of the nodes in the complex will be removed. By setting the fluff parameter, the MCODE can also detect overlapping clusters.

The CFinder algorithm is based on the clique percolation method [1]. CFinder finds all k-cliques of the original network in which k is an adjustable parameter, such that the larger the value of k, the higher the stringency during the identification of dense groups. As a result, smaller clusters with higher intra-cluster density are detected and the algorithm constructs a k-clique accessibility graph in which two k-cliques are adjacent if they share exactly k-1 nodes. From the connected component of the k-clique accessibility graph, CFinder detects the overlapping clusters. Furthermore, an intensity threshold I is introduced to include only the cliques whose product of edge weights products is greater than the threshold in the k-clique accessibility graph.

The Affinity Propagation algorithm (AP) finds clusters based on a random walk and passing messages between nodes [23]. A so-called preference parameter controls the likelihood of each node to be selected as an exemplar (i.e. representative of a cluster) by exchanging real-valued messages between all nodes. Next, the nodes are grouped with their most representative exemplar. Finally, the messages are exchanged between nodes iteratively until the algorithm converges and finds the high-quality group of exemplars and corresponding non-overlapping clusters.

The Detect Module from Seed Protein (DMSP) integrates PPI networks and microarray data to predict protein complexes [51]. It first assigns weights to the edges in the network based on gene-expression similarities of the given pair of proteins by utilizing a fuzzy c-means algorithm [60]. Then, DMSP starts with a seed protein and extends it by its most promising neighbors, which is called a “kernel”. This augmentation is based on multiple criteria, such as the number of neighbors, the weight of each connection, and the final subgraph. Finally, the kernel is expanded iteratively by adding its adjacent neighbors based on the same criteria and an extra one. The new criteria indicates that a kernel can be augmented by its adjacent neighbor, u, only if the u weight is less or equal to a specific percentage of the weighted degree of a given kernel.

The Clustering based on Maximal Clique (CMC) is another clique percolation-based algorithm [45], where CMC finds the maximal clique instead of detecting k-clique in the CFinder algorithm. CMC utilizes an iterative edge scoring method to weigh the interactions, which indicates the reliability of the protein interactions. It enumerates all maximal cliques in the network followed by a series of merging highly overlapped cliques to obtain the final complexes. The CMC has two parameters: overlap threshold and merging threshold. The overlap threshold determines when two cliques are highly overlapped, whereas the merge threshold decides how to proceed with the two highly overlapping cliques: the two cliques will be merged if the density of the overlapping part is greater than the merge threshold, otherwise the smaller clique will be discarded. The identified clusters have only clique topology, and their size is highly dependent on the parameters.

The PEWCC consists of two steps: pre-processing and finding protein complexes based on a local clustering coefficient [99]. Due to the availability of false-positive interactions in PPI networks, the PEWCC calculates the PE-score for each interaction and removes the edges with a reliability score lower than a given threshold r. The PE-score is calculated based on the probability that the neighboring nodes of the interacting proteins do not support the interaction between the two proteins. Next, the PEWCC calculates the clustering coefficient for each node and removes the nodes with the lowest degree until a core complex with three nodes is identified. This procedure is followed by expanding the core complex by nodes that interact with more than a given threshold of t% of the core nodes. Although PEWCC takes the noisiness of the PPI networks into account, it detects highly overlapped and repetitive clusters.

The ProRank+ is based on a ranking algorithm and has several steps, including pruning, filtering, ranking, finding protein complexes, and post-processing [30]. The pruning stage assigns a score to the interactions based on AdjustCD [45], a weighting procedure that iteratively calculates a score for each edge based on topological structure. The interactions with a score less than a given threshold are discarded. Next, ProRank + filters the proteins that act as a bridge, have a sparse neighborhood, and have at least one neighbor with significantly fewer interactions with other proteins. In the next step, the proteins are ordered decreasingly based on the ranking procedure. The protein complexes are formed by grouping the high-ranked proteins (i.e. essential proteins) and their neighbors as a cluster. The algorithm utilizes a merging threshold parameter to merge the protein complexes that share several essential proteins beyond a given threshold. Although ProRank + might remove false-positive edges, it does not consider false-negative edges.

The Discovering Protein Complexes based on Neighbor Affinity and Dynamic Protein Interaction Network (DPC-NADPIN) is a neighbor affinity-based algorithm [73]. The algorithm starts by ordering the nodes according to their local clustering coefficient. Next, nodes with clustering coefficient scores higher than a given threshold $T_{c}$, including their neighbors, initiate the core complexes. The procedure continues by expanding the clusters with their neighboring nodes per their neighbor affinity score. The expansion continues iteratively such that each time the neighbor node with the highest neighboring affinity score will be added to the corresponding cluster. The procedure terminates when the extension level reaches a recommended threshold of $T_{g}$. The final protein complex set is obtained after removing redundant clusters. Finding protein complex process in DPC-NADPIN does not define how to distinguish between protein complexes and functional modules dynamically. However, they integrate gene expression data with the PPI network to build dynamic PPI networks and then apply their algorithm to each temporal PPI network.

The identify Protein Complexes from weighted PPI networks using Mutual Important Interacting Partner relation (idenPC-MIIP) [89] predicts protein complexes in three steps. First, it finds MIIP for each node by defining mutually important neighbors on the weighted network. The parameter α is used to show to what extent the two neighbor proteins are mutually important to each other. Next, the seed node is a node with the highest degree, and all its MIIPs have formed a cluster c. In several rounds, the cluster c is expanded by its neighboring proteins based on specific rules, depending on whether the added protein is connected to the seed until there are no more proteins to be added. This is then identified as a cluster, and the procedure continues with the node of the next highest degree which does not belong to any of the generated clusters. Finally, the algorithm removes the clusters that are included in others or have only one node, and two clusters are merged if their overlap score is higher than the given threshold.

### 5.1 Cluster quality-based methods

The cluster quality-based approaches define a quality function and detect the clusters such that the maximum quality is obtained. The clusters are formed from different seeds via iteratively adding or removing nodes to gain their optimal quality.

The Restricted Neighborhood Search Clustering (RNSC) algorithm identifies protein complexes based on two cost functions and the algorithm has two main steps, (i) clustering and (ii) filtering clusters based on their functional similarity [39]. To predict protein complexes, RNSC starts with (random) clusters provided by the user as an input. The algorithm utilizes a naive cost function (simple integer-valued cost function) in the few initial steps. To refine the clusters, in each round, RNSC randomly moves nodes between clusters to improve the cost function. In the further steps, the algorithm upgrades to use a scaled cost function (more expressive real-valued cost function) until convergence. Finally, the clusters with size, density, and functional homogeneity lower than the given thresholds will be removed. Since RNSC is randomized, it returns different clusters in different executions.

The Module Inference by Parametric Local Modularity (miPALM) algorithm [101] combines the parametric local modularity measure and a greedy search to identify protein complexes. First, miPALM assigns weights to all interactions based on the number of common neighbors and node degrees. It then enumerates all triangles followed by ranking them based on triangle weights obtained by averaging pair-wise edge weights. Next, the miPALM repeatedly merges the top-ranked triangle with its immediate neighbor to maximize the local modularity until no additional neighbor leads to an increase in the local modularity. This procedure is then continued with a new top-ranked triangle. miPALM has two parameters α and δ; the former controls the background neighborhood size around a candidate complex, and the latter checks the density of the candidate complex. Finally, the small complexes are removed from the final set. The algorithm detects overlapping clusters, however it does not consider the effect of false-positive and false-negative interactions.

The Clustering with Overlapping Neighborhood Expansion (ClusterOne) algorithm aims to detect clusters with high cohesiveness [55]. The algorithm consists of three main steps. ClusterOne, iteratively, starts from a seed node with the highest degree. Then, a greedy procedure adds or removes nodes to detect clusters with high cohesiveness. Since the procedure for adding and removing nodes starts from multiple nodes, there is a possibility of finding overlapping clusters. In the second step, the algorithm quantifies the extent of overlap between pairs of clusters and merges them, where the overlap score is higher than a specified threshold. The overlap score calculates the number of common nodes between pairs of clusters to the power of two divided by the product of the total number of nodes in both clusters. Finally, the algorithm discards the clusters with a density below a given threshold or containing less than three nodes. ClusterOne incorporates the reliability of the protein interactions in its algorithm, and it finds overlapping clusters. However, it does not account for the effect of false-negative interactions, and it only predicts dense clusters.

The Core&Peel method [63] attempts to maximize the density of obtained clusters. In the initial phase, the algorithm computes the core decomposition of an original network where each node belongs to a maximal connected subgraph that all nodes have a degree of at least k. A node with the highest k-core is then selected as a seed. The induced subgraph of a selected node along with its neighbors, who are part of the same or greater k-core, should satisfy two criteria: the number of nodes in this subgraph should be greater than a pre-defined threshold q and have a density higher than a given value δ. Next, the peeling process iteratively removes nodes with a minimum degree until the density of the cluster is above or equal to the user-defined δ or the number of nodes drops below the threshold q. The final cluster set will be obtained after eliminating duplicates as well as clusters completely embedded in other clusters. The Core&Peel can detect overlapping clusters while it does not consider the noisiness of PPI networks in its algorithm.

The Inter-Module Hub Removal Clustering (IMHRC) algorithm identifies clusters based on the cohesiveness cluster quality measure in four steps [48]. The algorithm removes the top β% of the nodes with the highest degree (hub nodes) to eliminate false-positive interactions. In the second step, IMHRC predicts protein complexes with the same greedy procedure as ClusterOne accomplishes. The algorithm continues by inserting the top γ% of the removed hub nodes into clusters and checks whether adding them to the primary clusters will increase the cohesiveness quality measure or not. Next, the clusters with significant overlap above the specified threshold are merged. In the final stage, the clusters with a density below a fixed value (of 0.3) or consisting of fewer than three nodes are discarded. The IMHRC performs closely to ClusterOne since the procedure of finding the protein complexes from PPI networks is identical.

The last three approaches in this category, namely: Protein Complexes from Coherent Partition (PC2P) [58], Greedy Clustering Coefficient and its Variants (GCC-v) [57], and minimum CUt to detect Biclique spanned subgraphs as protein COmplexes (CUBCO) [59], formalize the protein complexes as biclique spanned subgraphs to include both sparse and dense complexes. As a result, they resolve the issues with community density and size observed in existing approaches. Moreover, these approaches cast the problem of protein complex prediction as a network partitioning into biclique spanned subgraphs, which is equivalent to the coherent network partition (CNP) problem [3]. The optimum CNP is obtained by removing a minimum number of edges that results in a network partition into biclique spanned subgraphs. This is shown to be an NP-hard problem [3], [4]. Thereby, the three approaches are based on parameter-free greedy heuristics (without provable approximation ratios for general graphs) that identify (sub)-optimal CNPs. Each method is explained in detail in the following.

Given a graph G, PC2P [58] determines a score for every node u that quantifies the quality of a biclique spanned subgraph in the second neighborhood of u, denoted by $N_{2}\left(u\right)$. Then, it selects the node with the smallest score and removes the biclique spanned subgraph in $N_{2}\left(u\right)$ from the graph as the first complex. The procedure is repeated as long as there are connected components in G.

GCC-v [57] is a family of greedy algorithms based on the concept of clustering coefficient and line graph. Given a graph G, the greedy algorithm determines a score for every node based on the clustering coefficient. Depending on whether the unweighted or weighted clustering coefficient is used to calculate the score for the nodes in the original or the line graph, the four different variants are obtained, namely: (i) clustering coefficient (CC), (ii) weighted clustering coefficient (WCC), (iii) overlapping clustering coefficient (OCC), and (iv) overlapping weighted clustering coefficient (OWCC). The greedy algorithm selects a node with the highest score and removes its neighbors along with the node itself from the graph. The next step updates the score of the nodes in the first neighborhood of the nodes in the identified cluster. This procedure is repeated as long as there are connected components in G.

Unlike the two previous approaches based on local graph properties, CUBCO [59] utilizes global properties to partition the network into biclique spanned subgraphs. The local algorithms utilize the local node properties, such as their first and second neighborhoods. In contrast, the algorithms based on global properties explore the whole graph at once. CUBCO iteratively finds the biclique spanned subgraph in a given graph G in three steps: (i) determine the complement of a graph G, i.e., $\overset{¯}{G}$, (ii) assign weights to the edges in $\overset{¯}{G}$ based on the degree-normalized number of paths of length three between the endpoint nodes of an edge in original graph G; (iii) iteratively find the global minimum cut of the edge-weighted graph $\overset{¯}{G}$ until all resulting components are biclique spanned.

### 5.2 Network embedding-based methods

Network embedding transforms nodes of a given graph G into a low dimensional space while preserving the structure and node/edge attribute affinity of the graph. In doing so, node similarity in the embedding space aims to provide a good approximation of the node similarity in the original graph. Therefore, it is important to find a mapping function f to transform the nodes into a d-dimensional space. After embedding, the new node space can be used with conventional machine learning methods as an input to solve several network analysis tasks, such as network clustering, link prediction, node classification, and network visualization [92]. There exist several mapping functions with a focus on preserving the topological structure of the original graph, such as DeepWalk [64], node2vec [28], and LINE [81]. There are other mapping functions, which try to preserve both topological structure and node/edge attribute affinity, such as MMDW [84], TADW [95], and AANE [32]. In general, the network embedding-based approaches first find the vector representation of nodes in low dimensional space. Next, they find the pair-wise similarity between the node vectors that are connected with an edge in the original network to make a new weighted adjacency matrix. Second, they use any clustering algorithm that considers the edge weight to predict protein complexes.

The GANE algorithm predicts protein complexes based on Gene Ontology (GO) attributed network embeddings [91]. The algorithm detects protein complexes using two main steps: (1) it transforms each protein to a vector representation by using a GO attributed PPI network via accelerated attributed network embedding (AANE) [32] mapping function. Hence, it preserves both the topological structure and node/edge attribute affinity of the graph. Then, it generates a weighted adjacency matrix based on the similarity of vector representations for each pair of nodes; (2) it utilizes a core-attachment structure to predict protein complexes. For this reason, the candidate cores are generated by using the clique mining method, and the core candidates are then ranked based on their densities on the weighted adjacency matrix. The attachments of a seed core are selected if the correlation score of a neighbor protein is larger than a given threshold θ. Finally, GANE returns the seed core and its attachments as a predicted protein complex.

The Complex Prediction algorithm based on Network Motif (CPNM) [62] predicts protein complexes through the embedding of network motifs. The algorithm has two main steps. First, it starts by finding network motifs followed by defining the role of each protein in every identified motif. The role of a protein is quantified by its degree in the PPI network. Therefore, two proteins are considered similar if they have the same role in different network motifs. With this, CPNM introduces a role matrix R with size n×m, where n is the number of proteins and m is the number of different roles, in which each entry $r_{\mathrm{ij}}$ illustrates the number of times the i ^th^ protein plays role j. Then, the feature matrix is obtained by concatenating all role matrices from all the network motifs and is then normalized. Each row of the normalized feature matrix and its summation are referred to as NMVector and NMWeight. In the second step, the CPNM procedure receives the original PPI network, NMVector, and NMWeight as input arguments to predict protein complexes by a neighborhood search approach. Therefore, CPNM selects a seed node and iteratively adds neighboring nodes based on three constraints: (1) the attached node should be the neighbor of the nodes in the complex, (2) the Manhattan distance between the NMVector of two nodes should be the lowest between all the adjacent nodes, and (3) by augmenting a complex with a node, the average weight of the complex should not be lower than the predefined threshold.

Detecting Protein Complexes from protein-protein interaction networks via Multi-level Network Embedding (DPCMNE) [53] detects protein complexes by utilizing multi-level network embeddings, which preserves global as well as local topological information. The DPCMNE method hierarchically compresses the PPI network by adopting the Louvain clustering algorithm [9] to obtain multi-level smaller PPI networks $G^{0},G^{1},\cdots ,G^{L}$. Then, DPCMNE employs DeepWalk [64] to every compressed PPI network to generate protein embeddings $H^{0},H^{1},\cdots ,H^{L}$. The final embedding of each protein is obtained by concatenating the embeddings from all compressed PPI networks. The pairwise cosine similarity of the interacted protein vectors is calculated to obtain the new weighted adjacency matrix of the original PPI network. In the next step, DPCMNE uses a similar approach to the GANE method to predict protein complexes based on the core-attachment structure. Therefore, it first finds all the cores and ranks them based on their densities, which considers the local and global properties. Then, it selects a core with the highest score as a core seed to augment it with suitable attachment proteins by calculating a connectivity function. The neighbor protein will be attached to the core seed if its connectivity score is greater than a given threshold λ. Finally, DPCMNE returns the core seed and its attachment as a predicted protein complex.

## 6 Evaluation metrics

There exist twelve well-established metrics to evaluate the performance of protein complex prediction approaches by comparing the reference protein complexes from gold standards with predicted complexes, that is sensitivity, positive predictive value, accuracy, separation [12], fraction match, maximum matching ratio [55], precision, recall, and F-measure [45], as well as precision^+^, recall^+^, and F-measure^+^ [49]. Each of these metrics has its advantages and disadvantages which are critically assessed in [49].

Let $R=\{r_{1},r_{2},{\cdots ,r}_{n}\}$ and $P=\{p_{1},p_{2},{\cdots ,p}_{m}\}$ be the set of reference and predicted protein complexes, respectively. A contingency table T is constructed with n rows representing complexes in R, and m columns denoting predicted complexes in P. The entry $t_{i,j}$ represents the number of shared proteins between $r_{i}$ and $p_{j}$. The positive predictive value (PPV), sensitivity (SN), accuracy (ACC), and separation (SEP) are defined as:

(1) $$\mathrm{PPV}=\frac{\sum_{j}\max_{i}\left(t_{i,j}\right)}{\sum_{j}\sum_{i}t_{i,j}},$$

(2) $$\mathrm{SN}=\frac{\sum_{i}\max_{j}\left(t_{i,j}\right)}{\sum_{i}t_{i,j}},$$

(3) $$\mathrm{ACC}=\sqrt{\mathrm{PPV}\times SN},$$

(4) $$\mathrm{SEP}=\sqrt{\frac{1}{\mathrm{nm}}\sum_{i}\sum_{j}\left(\frac{t_{i,j}}{t_{.j}}\times \frac{t_{i,j}}{t_{.i}}\right)\times \sum_{j}\sum_{i}\left(\frac{t_{i,j}}{t_{.j}}\times \frac{t_{i,j}}{t_{.i}}\right)}.$$

The overlap score between the pair of protein sets $r_{i}$ and $p_{j}$ is given by [7]:

(5) $$OS\left(r_{i},p_{j}\right)=\frac{{|r_{i}\cap p_{j}|}^{2}}{|r_{i}\left|\right|p_{j}|}.$$

If $OS\left(r_{i},p_{j}\right)\geq \theta$, $r_{i}$ and $p_{j}$ match to each other. The value of θ is varied in different studies. For instance, in [55], the θ value is set to 0.25 while in [77] is equal to 0.5.

The fraction match (FRM) calculates the ratio of matched predicted protein complexes to the number of reference complexes. The maximum matching ratio (MMR) is based on a bipartite graph, in which the vertices in each partition correspond to reference and predicted protein complexes, individually, and the edges are weighted by the overlap score between the two partitions. Then, MMR is given by the ratio of the sum of the weight of the maximal matching edges to the number of reference complexes.

The precision, recall, and F-measure are based on the matched predicted protein complexes and defined as:

(6) $$\mathrm{Precision}=\frac{\left|p_{i}\in P|\exists r_{j}\in R,p_{i}matchesr_{j}\right|}{\left|P\right|},$$

(7) $$\mathrm{Recall}=\frac{\left|r_{i}\in R|\exists p_{j}\in P,p_{j}matchesr_{i}\right|}{\left|R\right|},$$

(8) $$F-measure=\frac{2\times Precision\times Recall}{\mathrm{Precision}+Recall}.$$

The precision^+^ and recall^+^ are given by $\frac{N_{P}^{+}}{\left|P\right|}$ and $\frac{N_{r}^{+}}{\left|R\right|}$, respectively. Whereby, $N_{P}^{+}$ and $N_{r}^{+}$ are defined as:

(9) $$N_{P}^{+}=\left|{\{p}_{i}\in P\left|\exists r_{j}\in R,OS\left(p_{i},r_{j}\right)\geq \theta ,\left(p_{i},r_{j}\right)\in Match\left(P,R,\theta \right)\right\}\right|,$$

(10) $$N_{r}^{+}=\left|{\{r}_{j}\in R\left|\exists p_{i}\in P,OS\left(p_{i},r_{j}\right)\geq \theta ,\left(p_{i},r\right)\in Match\left(P,R,\theta \right)\right\}\right|.$$

The Match(P,R,θ) function obtains the set of edges by employing a maximum non-weighted matching algorithm on the bipartite graph that has reference complexes on one side and the matched predicted complexes on the other side. The F-measure^+^ is calculated the same way as the original F-measure but with precision^+^ and recall^+^.

To summarize the twelve performance measures and to enable its visualizations, two composite scores are defined. The first composite score is given by the sum over MMR, FRM, SEP, ACC, F-measure [55], [13], [87], [57], [57], [59], and the second one is the sum over MMR and F-measure^+^ across different threshold values 0≤θ≤1 [49], [57], [57], [59].

To further evaluate the predicted protein complexes concerning biological relevance, two different types of analyses can be performed, that is: Over Representation Analysis (ORA) [11] and GO semantic similarity [35]. GO is a hierarchical controlled biological vocabulary that estimates the functional similarity of gene products, relating to three categories: (i) Molecular Function (MF), (ii) Biological Process (BP), and (iii) Cellular Component (CC).

ORA is one of the commonly used approaches to determine whether a set of genes, i.e. proteins in a predicted protein complex, is overrepresented by known biological functions or processes more than what we expected by chance. To this end, the p-value is calculated by hypergeometric distribution as follows:

(11) p-value=1-∑i=0k-1FiV-FC-iVC,

Where V contains all proteins in the PPI network, F is a functional group with annotated genes, and C is a predicted protein complex that includes k proteins. The smallest p-value is selected over all possible functional groups for each predicted protein complex. Therefore, lower ORA indicates that the predicted protein complex is enriched by proteins from the same functional group, hence, it is more likely to be a true protein complex. By defining a threshold on the statistical significance level one can count the number of overrepresented predicted protein complexes whose ORA value is lower than a given threshold to evaluate and compare different algorithms. Although ORA is a well-established approach, it suffers from several shortcomings. ORA is determined by the assumption of gene-gene independence, while this is not valid biologically [61]. Moreover, ORA depends on a set of differentially expressed genes as input where all genes are treated equally irrespective of their magnitude of differential expression [38]. Finally, determining an arbitrary threshold might affect the downstream analysis result [50].

The GO semantic similarity [16] determines the functional similarity of two given proteins based on two different measurements: (i) information content-based (IC) and (ii) graph-based. The IC-based approaches calculate the semantic similarity based on the information content of their closest common ancestor, i.e., most informative common ancestor (MICA). The Resnik [69], Rel [71], Lin [43], and Jiang [35] methods are IC-based approaches, to name a few. The graph-based methods determine semantic similarity by employing the topological structure of the GO graph. One of the widely used methods in this category is the Wang method [86]. Regardless of which category is employed, the GO semantic similarity is determined for all pairs of proteins in every predicted protein complex for different categories of GO individually. The final value for each complex can be obtained by calculating the minimum, maximum, or median of its whole pairs. The distribution of the GO semantic similarity overall predicted protein complexes can be used to compare different approaches. This assessment can support the hypothesis that a protein complex includes proteins with similar molecular functions and involved in the same cellular component. Despite the popularity of the GO semantic similarity approaches, they suffer from limitations, such as not being able to handle identical annotations, and similar to ORA, show strong bias toward well-annotated proteins [94].

The ORA and GO semantic analyses have their advantages and disadvantages. Both analyses evaluate the predicted protein complexes biologically and give an overview of how each computational approach performs regarding biological significance. However, the required information to calculate them is limited. The number of predicted protein complexes and the number of proteins in each of them can affect the two measurements. In [57], the distributions of GO semantic similarity of three GO categories are computed and shown for predicted protein complexes across several network clustering algorithms. This investigation suggests that the approaches detecting a smaller number of protein complexes illustrate narrower distributions for GO semantic similarities.

### 6.1 Biological relevance of protein complexes in the available gold standards

Here, we analyzed the biological significance of complexes in the gold standards of three species, *E. coli*, *S. cerevisiae*, and *H. sapiens*, by calculating the median GO semantic similarity of their reference complexes. It is expected that the reference complexes would achieve high semantic similarity values; however, this is not the case. In the case of *E. coli*, the number of proteins, protein complexes, and protein complexes with more than three proteins is lower in the gold standard of metabolic protein complexes than Ecocyc, by ∼ 57%, ∼45%, ∼53%, respectively. As a result, Ecocyc contains larger protein complexes and covers more proteins than Metabolic protein complexes.

In *S. cerevisiae*, the CYC2008 and SGD gold standards contain almost the same number of protein complexes with more than three proteins, SGD includes two complexes fewer than CYC2008. The number of proteins and protein complexes is smaller in SGD compared with CYC2008 by ∼ 27%, and ∼ 26%, respectively.

**Fig. 3:**
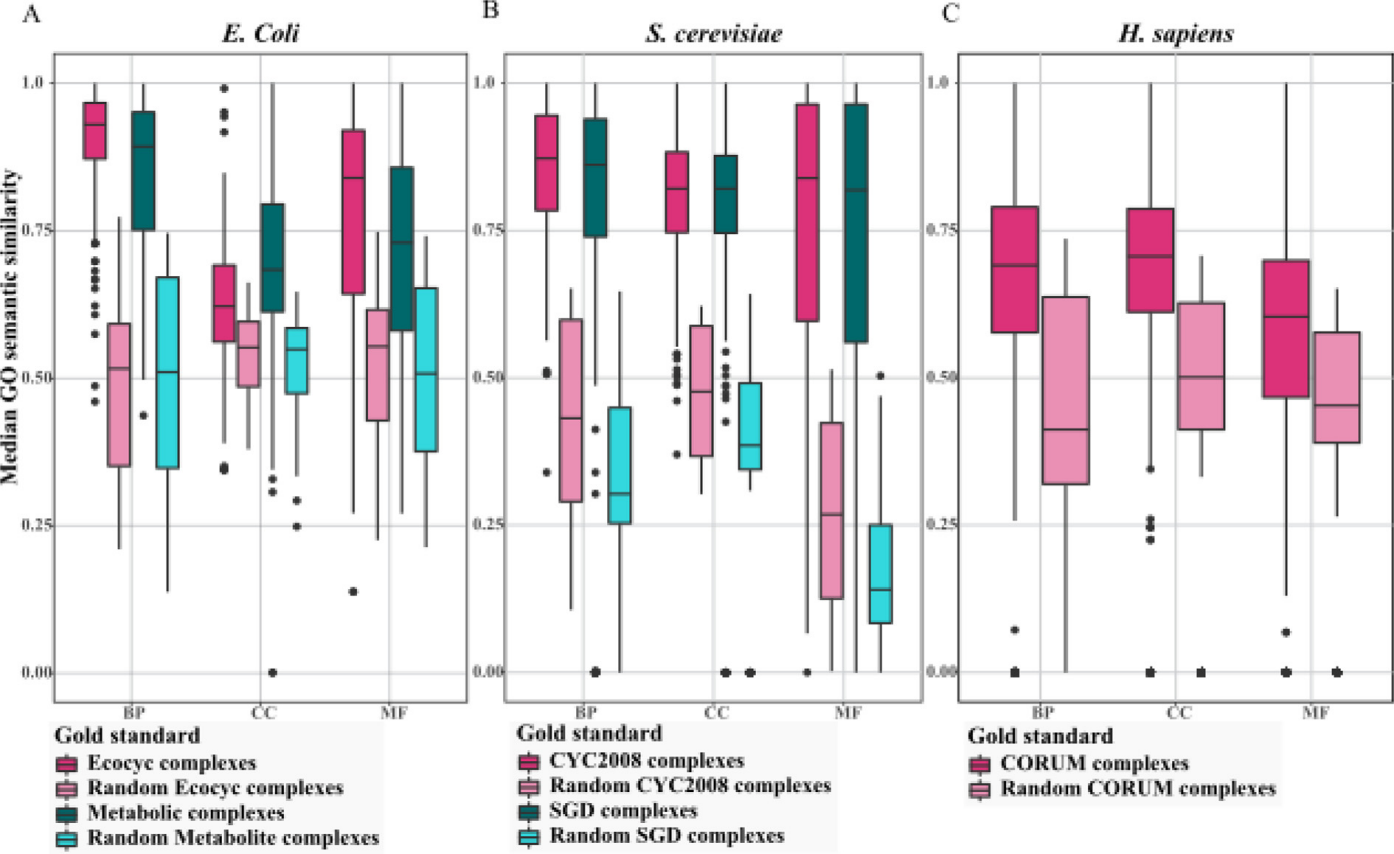
**GO semantic similarity analysis of protein complexes of gold standards.** The distribution of median GO semantic similarity of reference complexes is compared with the randomly generated complexes from altogether five gold standards for three species: (A) *E. Coli*, (B) *S. cerevisiae*, and (C) *H. Sapiens* and their randomized variants.

The GO semantic similarity across BP and MF categories of GO in *H. sapiens* is less than the other two species, on average by ∼ 23% and ∼ 36%, respectively, and on average in GO:CC, *H. Sapiens* shows semantic similarity value close to that in the other two species. To compare between categories of GO, it appears that data on GO:CC is more incomplete than the other two categories in *E. Coli* and *S. cerevisiae*. While GO:BP obtains better values in *E. Coli* and *S. cerevisiae* and is comparable with GO:CC in *H. sapiens* (Fig. 3).

We further extend the analysis by providing the estimates for the expected values of three categories of GO semantic similarity across the three species when applied to randomized complex sets. In order to randomize a complex set while maintaining the size distribution of the complexes, first, a list of proteins for each gold standard is created by joining all reference complexes. To this end, the list is shuffled based on the Fisher-Yates shuffle [21], and divided into random complexes such that the size of the original reference complexes is preserved. The value of median GO semantic similarity of three categories is computed for the generated random sets. Finally, this procedure is repeated 50 times, to estimate the expected GO semantic similarity value for each category. We estimated these values for each gold standard across three species (Fig. 3). We concluded that the median of the GO semantic similarity over randomized gold standards is lower than the median obtained from the protein complexes in gold standards, in each of the three species. More specifically, for both gold standards of *E. Coli*, on average, the median GO semantic similarity of expected value is lower by ∼ 43.5%, ∼13%, and ∼ 30.5% than the median GO semantic similarity of true complexes for three categories, BP, CC, and MF, respectively. In the case of *S. cerevisiae*, on average, the median GO semantic similarity of the expected value of BP, CC, MF is lower by ∼ 57.5%, ∼47.5, and ∼ 74% than the median GO semantic similarity of reference complexes of CYC2008 and SGD. Lastly, for *H. sapiens*, the expected GO semantic similarity values are only lower by ∼ 38%, ∼28%, and ∼ 26% than the corresponding values for reference complexes in the CORUM, concerning BP, CC, and MF, respectively.

## 7 Comparative evaluation of protein complex prediction methods

Here, we compared the performance of eighteen state-of-the-art approaches for protein complex prediction of which eight belong to node affinity-based, nine to cluster quality-based, and one to network embedding approaches. To facilitate a fair comparison, the approaches are selected based on two criteria, (i) the public availability of executable code and implementations and (ii) that the method does not rely on any additional biological knowledge or data. Therefore, MCL [22]), MCODE [7], CFinder [1], AP [23], CMC [45], PEWCC [99], Prorank+ [30], and DPC-NADPIN [73] are selected from the node affinity-based category; ClusterOne [55]), Core&Peel [63], IMHRC [48], PC2P [58], CC [57], WCC [57], OCC [57], OWCC [57], and CUBCO [59] from the cluster quality-based category; and DPCMNE [53] from network embedding-based category. For the parameter(s)-dependent approaches (see Table 3), we used the default parameter values as suggested in the corresponding original studies.

From the GO semantic similarity analysis of gold standards of different protein complexes for different species, it can be concluded that more accurate protein complexes are included in the gold standards for the species *S. cerevisiae* (CYC2008 [66] and SGD [31], see Fig. 3). While both CYC2008 and SGD show similar results, we decided to use CYC2008 for our comparison, since more protein complexes are included to compare with SGD. To conduct a comparative analysis of different approaches, we used four PPI networks of *S. cerevisiae* namely, Collins [17], [18], Gavin [24], KroganCore [42], and KroganExt [42]. Consequently, to assess the performance of these approaches, we used CYC2008 as the gold standard.

**Fig. 4:**
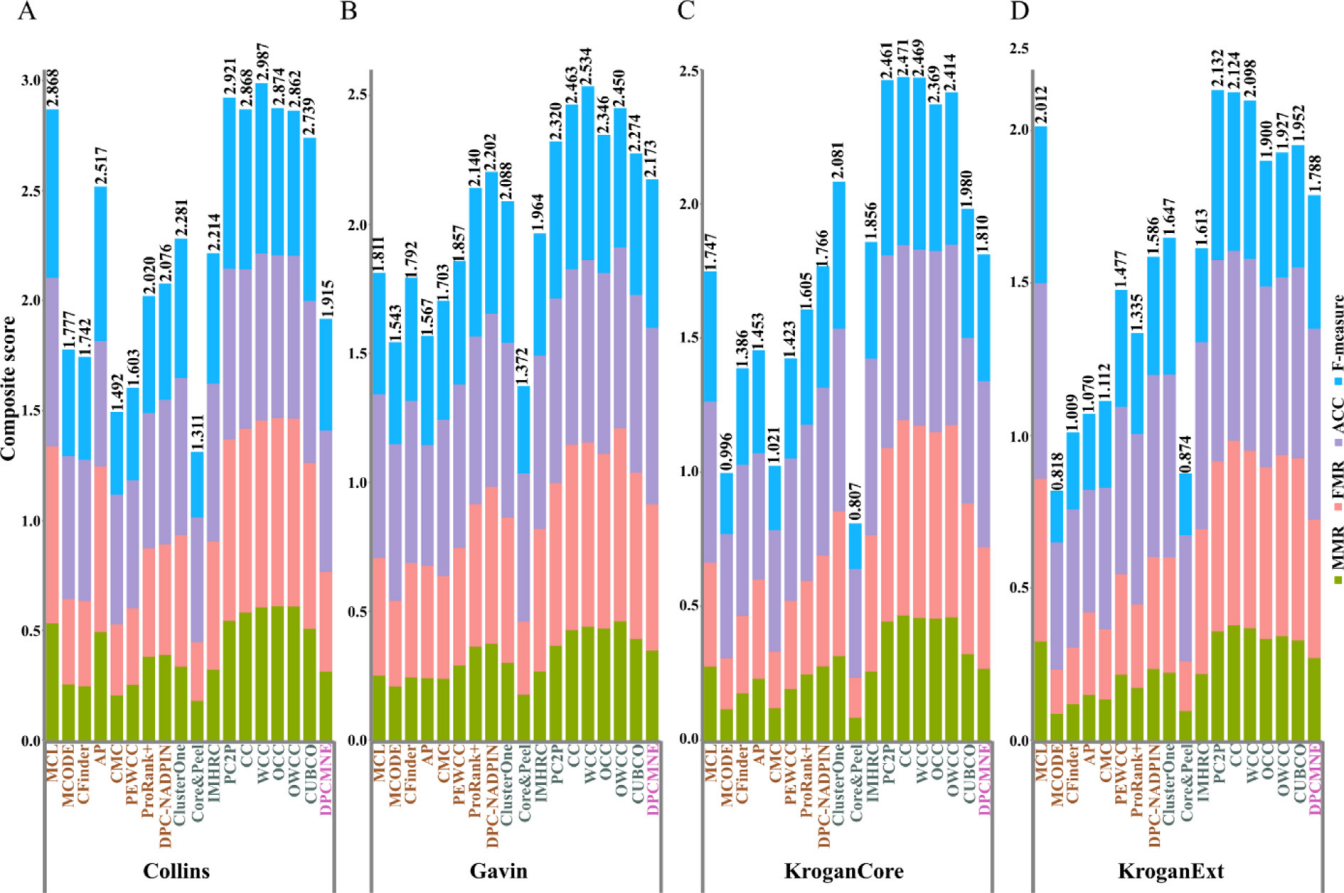
**Comparative analysis of approaches for prediction of protein complexes.** Eighteen state-of-the-art approaches are applied on four PPI networks of *S. cerevisiae*, which are (A) Collins, (B) Gavin, (C) KroganCore, and (D) KroganExt. The predicted clusters from different approaches are compared with protein complexes in the gold standard CYC2008. The comparative analysis is conducted with respect to a composite score, which is the summation of four performance measures, maximum matching ratio (MMR), fraction match (FRM), accuracy (ACC), and F-measure. Eighteen approaches are ordered first by their categories, node affinity-based (in brown), cluster quality-based (in green), and network embedding-based (in pink). Second, the methods in each category are ordered by the year of publication. The result indicates that the cluster quality-based methods, more specifically, those that model a protein complex as a biclique spanned subgraph outperformed the others. (For interpretation of the references to color in this figure legend, the reader is referred to the web version of this article.)

To this end, we determined twelve well-established performance measures, including maximum matching ratio (MMR), fraction match (FRM), separation (SEP), sensitivity (SN), Positive predictive value (PPV), accuracy (ACC), precision, recall, F-measure, precision^+^, recall^+^, and F-measure^+^ (see Section 5 “Evaluation metrics”). The range of the given metrics is between zero and one, the higher value indicates the better performance. Moreover, we calculated a composite score, which is the sum over MMR, FRM, ACC, and F-measure for all eighteen approaches. The overlapping score θ is set to 0.5 as suggested by these studies [77], [57], [57], [59]. Supplementary Table 1 shows the overall performance of all eighteen contenders with respect to the twelve performance measures. Fig. 4 illustrates the results across the four PPI networks concerning the composite score.

Overall, based on comparative analysis, the approaches belonging to cluster quality-based achieved better composite score than the approaches in the other two categories across all PPI networks. While MCL from node affinity-based methods ranked first in its category and showed results on par with the cluster quality-based approaches for Collins and KroganExt PPI networks. Moreover, the methods based on biclique spanned partitioning of the network, PC2P, CC, WCC, OCC, OWCC, and CUBCO indicate similar and consistent results across all PPI networks and exhibited the highest performance. More precisely, PC2P obtained the highest accuracy for all PPI networks and outperformed the other approaches regarding separation, F-measure, and F-measure + for three out of four PPI networks, Collins, KroganCore, KroganExt. Likewise, WCC achieved the highest positive predictive value across all PPI networks. Therefore, not surprisingly, WCC ranked first in Collins and Gavin PPI networks, while PC2P and CC outperformed the other contenders in KroganExt and KroganCore, respectively concerning the composite score (see Supplementary Table 1).

## 8 Summary and outlook

A primary goal of biology is to understand how the different components of cells function as a system to perform diverse tasks. Proteins, as a key component of the cell, participate in various molecular functions and biological processes, and more importantly, they do not act alone but interact with each other to form macromolecular components, i.e. protein complexes. Therefore, the study of protein complexes plays an important role to understand the cellular hierarchy and molecular mechanism.

The increasing availability of high-throughput data facilitates the *in silico* study of protein complexes through the construction of protein–protein interaction (PPI) networks. In the past decades, several computational approaches have been proposed to address the problem of predicting protein complexes given a PPI network as input. The computational approaches show increasing improvement of performance over time which has led to the detection of more accurate protein complexes. However, there is still room for improvement in this field, as pointed out in the following.

### 8.1 Critical assessment of existing PPI networks and protein complex gold standards

Since PPI networks are the input of protein complex prediction algorithms they play an important role in their performance. However, these networks are still incomplete and noisy [88] and include false-positive as well as false-negative interactions. The information on non-interacting proteins (NIPs) could be a great advantage for algorithms in the field of protein interaction detection and the evaluation of the false-positive rate of PPIs in PPI networks. Negatome [8] is a database that includes 6532 PPIs that are unlikely to physically interact with each other. To this end, we selected BioGRID [78] as one of the pioneers in collecting PPIs, including 798,241 interactions of *H. Sapiens* in its recent version (4.4.206), and we compared BioGRID with PPIs in Negatome. First, we converted the protein identifiers in Negatome, from UniProt to gene name, and with this, we could only retrieve the gene name for 5808 PPIs in Negatome, of which 965 PPIs are also presented in the BioGRID PPI network. On the other hand, due to the limitations of high-throughput approaches, different types of false-negative interactions are also present in PPI networks, such as week transient interactions [82]. In conclusion, future studies can benefit from the set of non-interacting proteins to preprocess the input PPI network. Moreover, it can be utilized as a negative set in supervised link prediction algorithms.

Another issue with current PPI networks is that most of the existing data are largely static, providing only limited to no insights into the dynamics of cellular activity [36]. Therefore, understanding the dynamic nature of cellular processes remains a difficult task. Proteins are not an exception to the dynamics that take place at a molecular level; they associate and disassociate with each other at different time scales and in various cellular compartments to execute specific processes. Therefore, it is important to unravel the temporal complexity of PPI networks to be able to detect not only static, permanent protein complexes but also transient ones. To this end, one can compile time-series gene expression data and protein abundances along with protein sequences to bring the dynamics of PPI networks into the analyses and prediction of protein complexes. Several efforts have been made on assigning dynamic weights to PPIs and constructing dynamic PPI networks [101], [14]. However, there is still room to improve the quality and availability of these PPI networks across different species.

In addition, the gold standards include different small subsets of the proteins in the existing PPI networks. For instance, both Babu and Cong PPI networks of *E. Coli* (Table 1), on average, share ∼ 27% and ∼ 17% of their proteins with Ecocyc and Metabolite gold standard, respectively. While Ecocyc and Metabolite gold standards share, on average, ∼57% and ∼ 67% of their proteins with Babu and Cong PPI networks, respectively. This results in low coverage of reference complexes from protein complex prediction algorithms.

### 8.2 Protein complex prediction algorithms

Computational approaches complement the experimental methods to detect protein complexes from PPI networks. Several computational approaches have been proposed to date, of which we summarized 21 state-of-the-art approaches in this study along with their advantages and disadvantages. We further evaluate the performance of 18 out of 21 approaches on four PPI networks. The result illustrates that cluster quality-based methods outperformed the other two categories. More precisely, MCL from the node affinity-based group outperformed the other contenders in the same group. While PC2P, GCC-v, and CUBCO from the cluster quality-based category outperformed the other contenders from three categories in all cases.

One aspect that most of the approaches have in common is to find a highly connected region as a protein complex in PPI networks. In addition, many approaches mainly find large complexes and eliminate small, predicted clusters. However, recent studies concluded that protein complexes are dense as well as sparse, and they can be small, consist of two proteins, as well as large, with more than three proteins. These limitations have been addressed in (refs Omranian2021, Omranian2021a, Omranian_CUBCO) by casting the problem of protein complex prediction into biclique spanned partitioning of the network. However, in the recent category of protein complex prediction algorithms, network embedding-based approaches, after integrating multiple data into the network by constructing a weighted adjacency matrix of the original network, employ core attachment methods to detect final protein complexes. Therefore, the approaches in this category still suffer from predicting large as well as dense protein complexes.

Another issue is that different approaches heavily depend on multiple parameters, which render it difficult to interpret the predicted protein complexes. Depending on which PPI network, protein complex gold standard, and performance measure are used, the algorithms predict different sets of protein complexes. This problem is even worse in the case of network embedding-based approaches since they must not only set the parameters but also the hyperparameters to find the optimal set of protein complexes.

Moreover, most algorithms utilize different metrics to score the protein interactions and remove those with a score below a given threshold. However, they did not consider a way to bring the false-negative interactions into the PPI networks except CUBCO [59], which utilizes a link prediction algorithm. This is an important issue and should be considered in future studies.

Finally, as mentioned earlier, PPIs play a significant role in molecular functions and biological processes, and they can contribute to our understanding of cellular activities. Due to the labor-extensive and time-consuming experimental approaches, several computational methods have been developed to facilitate the prediction of PPIs (refs Patel2017, Wang2020). It is possible to improve the performance of link prediction algorithms by utilizing protein complex prediction algorithms in such a way that, first, the protein complex prediction algorithms cluster proteins with similar structures or attributes into the same group, and then several similarity measures can be used to compute the probability of interactions between proteins in the same group. These ideas provide directions that can be explored in future studies.

## CRediT authorship contribution statement

**Sara Omranian:** Conceptualization, Writing – review & editing. **Zoran Nikoloski:** Conceptualization, Writing – review & editing. **Dominik G. Grimm:** Conceptualization, Writing – review & editing, Supervision, Funding acquisition.

## Declaration of Competing Interest

The authors declare that they have no known competing financial interests or personal relationships that could have appeared to influence the work reported in this paper.
